# Pentraxin-3 as a Biomarker in Diabetes Mellitus: Insights into Inflammation, Vascular Complications, and Modulation by Antidiabetic Medications

**DOI:** 10.3390/biomedicines13040891

**Published:** 2025-04-07

**Authors:** Roxana-Cristina Dobriceanu, Andreea Daniela Meca, Ianis Kevyn Stefan Boboc, Liliana Mititelu-Tartau, Mihaela Simona Naidin, Adina Turcu-Stiolica, Maria Bogdan

**Affiliations:** 1 Doctoral School, University of Medicine and Pharmacy of Craiova, 200349 Craiova, Romania; dobriceanuroxana@yahoo.com; 2 Department of Pharmacology, Faculty of Pharmacy, University of Medicine and Pharmacy of Craiova, 200349 Craiova, Romania; andreea_mdc@yahoo.com (A.D.M.); bogdanfmaria81@yahoo.com (M.B.); 3 Department of Pharmacology, Faculty of Medicine, ‘Grigore T. Popa’ University of Medicine and Pharmacy, 700115 Iasi, Romania; 4 Department of Pharmaceutical Marketing and Management, Faculty of Pharmacy, University of Medicine and Pharmacy of Craiova, 200349 Craiova, Romania; mihaela.subtirelu@yahoo.com (M.S.N.); adina.turcu@gmail.com (A.T.-S.)

**Keywords:** pentraxin-3, biomarker, diabetes mellitus, inflammation, antidiabetic drugs

## Abstract

Diabetes mellitus (DM) is a multifactorial metabolic disorder associated with systemic inflammation and vascular complications. Pentraxin-3 (PTX3) has emerged as a key biomarker of inflammation and endothelial dysfunction in DM. We aimed to examine the role of PTX3 in DM and assesses the impact of pharmacological interventions on its expression. The review included studies analyzing PTX3 modulation by antidiabetic therapies, such as sodium-glucose cotransporter-2 inhibitors (SGLT-2i), glucagon-like peptide-1 agonists (GLP-1a), and dipeptidyl peptidase-4 inhibitors (DPP-4i), as well as the effects of lifestyle interventions. Clinical and experimental studies demonstrated a strong correlation between PTX3 levels and DM progression. Elevated PTX3 levels were associated with diabetic complications, including nephropathy, retinopathy, and cardiovascular diseases. Antidiabetic drugs showed differential effects on PTX3 expression, with GLP-1a and DPP-4i significantly reducing PTX3 levels, while SGLT-2i displayed a paradoxical increase. Lifestyle interventions, including dietary modifications and weight loss, yielded inconsistent effects, suggesting genetic and metabolic factors influence PTX3 regulation. While pharmacological therapies, particularly GLP-1a and DPP-4i, demonstrate anti-inflammatory effects, further research is needed to standardize PTX3 measurement and explore its potential as a therapeutic target. Personalized treatment strategies incorporating genetic profiling may optimize inflammation control and disease management in DM patients.

## 1. Introduction

Diabetes mellitus (DM) represents a multifactorial metabolic disorder marked by persistent hyperglycemia, which occurs either due to a lack of insulin (type 1 diabetes, T1DM) or to insulin resistance combined with insufficient insulin production (type 2 diabetes, T2DM) [1,2,3]. Excessive glucagon secretion can further worsen hyperglycemic control and mitochondrial dysfunction, therefore generating lipodystrophies and activating inflammatory signalization [3,4].

In 2019, 463 million adults were diagnosed with DM, contributing to 4.2 million deaths [2]. By 2021, the prevalence in adults was 10.5%, and in 2022, 14% of adults were affected worldwide, with treatment coverage remaining low in low- and middle-income countries. It is estimated that the number of DM diagnoses will grow to 12.2% by 2045, which means approximately 700 million new cases [2,5,6,7]. T1DM incidence in children is increasing by 3–4% per year, and approximately 84% of individuals living with T1DM are adults [8]. These trends emphasize the urgent need for improved prevention, early diagnosis, and better access to care [2].

As an autoimmune condition, T1DM involves the progressive destruction of pancreatic β-cells, resulting in both diminished insulin secretion and tissular glucose sensitivity [2,9]. T2DM accounts for 90–95% of diabetes cases, typically developing later in life due to reduced insulin sensitivity in muscle, liver, and adipose tissue, and is often accompanied by a higher proinsulin-to-insulin ratio and impaired response to non–glucose stimuli [1,2,9]. Gestational DM is associated with an increased risk of adverse outcomes for both the mother and newborn and represents the only transitory form of diabetes as the women can recover after pregnancy, while latent autoimmune diabetes in adults shares features with T2DM yet it is marked by pancreatic autoantibodies and an earlier need for insulin therapy [1,9].

Chronic hyperglycemia disrupts the metabolism of carbohydrates, fats, and proteins, leading to microvascular complications such as nephropathy, retinopathy, and peripheral neuropathy affecting both autonomic and peripheral nerves. Macrovascular complications can also appear and include the following: coronary heart disease, myocardial infarction, atherosclerosis, heart failure, and stroke [1,2,7,10]. Visceral obesity, common in T2DM, further exacerbates microvascular dysfunction in several organs, including the brain [11]. Moreover, non-alcoholic steatohepatitis has been noted as one of the most frequent complications in individuals with T2DM [9]. A recent classification divides these complications into three categories: vascular, parenchymal, and hybrid [12].

Treatment of T2DM employs several drug classes with distinct mechanisms: biguanides, sulfonylureas (SFN), thiazolidinediones (TZD), glucagon-like peptide-1 agonists (GLP-1a), dipeptidyl peptidase-4 inhibitors (DPP-4i), sodium–glucose transport protein 2 inhibitors (SGLT-2i). Less widely used classes of antidiabetic drugs are meglitinides (GLN) and alpha-glucosidase inhibitors (AGi) [13,14].

Recent studies indicate that SGLT-2i and GLP-1a also improve metabolism, facilitate weight loss, lower blood pressure, and confer renal protection, even in individuals without DM, possibly via reduced oxidative stress, enhanced mitochondrial function, and activation of various tissue-specific anti-inflammatory mechanisms [12,15]. Therefore, various antidiabetic drugs offer supplementary protection against specific complications.

The pentraxin (PTX) superfamily represents an evolutionarily conserved, cyclic multimeric protein family, which consists of pentameric structured proteins that include a common C-terminal domain with a conserved eight-aminoacid motif (His–x–Cys–x–Ser/Thr–Trp–x–Ser, where x represents any amino acid) [16,17,18,19,20]. Members possess a 203-aminoacid PTX domain attached at the carboxyl-terminal and are classified into two subgroups: short (classical) proteins, and long (fusion) proteins [17,18,21,22]. The short and most studied PTXs include C-reactive protein (CRP) and serum amyloid P (SAP/PTX2), which are acute-phase proteins produced by the liver in response to interleukin-6 (IL-6); these proteins can increase dramatically during inflammation and have proven efficient as markers for infections, cardiovascular diseases (CVD), autoimmune disorders, and cancers [18,20,21]. Long PTXs—comprising neuronal pentraxins (NPTX1 and NPTX2), pentraxin-3 (PTX3), and pentraxin-4 (PTX4)—act as soluble pattern recognition molecules and play a critical role in both innate and humoral immune responses and the inflammatory cascade [19,20,22].

The scope of this review is to correlate the impact of PTX3 in DM and progression of complications, as well as antidiabetic drugs’ influence, considering that inflammatory pathways are pillars in the evolution of this metabolic disease.

## 2. PTX3

PTX3 is a 381-aminoacid glycoprotein with a 17-aminoacid signal peptide. It features a unique N-terminal domain, composed of four helices (with three adopting a coiled conformation) and a C-terminal domain characterized by a hydrophobic core stabilized by two antiparallel β-sheets in a β-jelly roll topology with an associated α-helix [16,17,19,21]. Its main function involves binding to C1q via the C-terminal domain, thereby activating the classical complement cascade and promoting the clearance of pathogens and damaged cells [16,20,23]. Initially identified in human umbilical vein endothelial cells and FS-4 fibroblasts in the 1990s, PTX3 is now known to be expressed in smooth muscle cells, myeloid dendritic cells, epithelial cells, and tumor cells [22,24,25]. It functions as a soluble pattern recognition receptor and is produced locally by various cell types, including mononuclear phagocytes, fibroblasts, granulosa cells, mesangial cells, endothelial cells, and adipocytes, with its expression regulated by micro-RNAs, cytokines, transcription factors, and various drugs [17,22,24,25,26]. During inflammation, vascular production of PTX3 serves as an endothelial regulator in thrombosis and ischemic conditions, partly through binding angiogenic fibroblast growth factor-2 to inhibit angiogenesis and restenosis [25,27].

**Figure 1:**
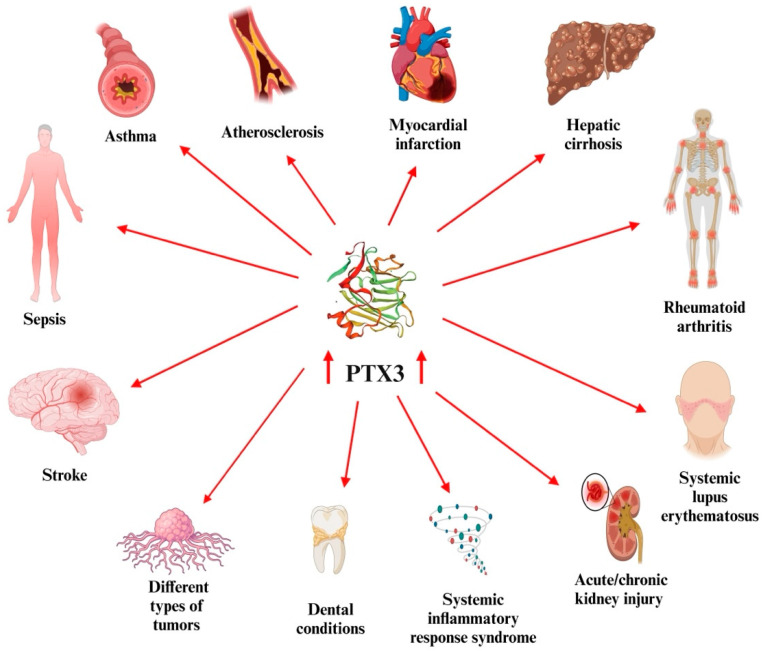
Various disorders associated with elevated PTX3 levels. (Created with BioRender.com, accessed on 1 March 2025).

Elevated PTX3 levels have been associated with greater disease severity and mortality in diverse conditions (Figure 1); it is overexpressed in different types of tumors (e.g., glioma, liposarcoma, melanoma, and prostate/breast/hepatic/lung cancer) and latest research has shown that PTX3 may play dual roles as a multifunctional protein in tumor biology depending on the context [17,22,28,29,30,31,32,33,34,35].

Different studies compared PTX3 values to other biomarkers in several conditions. PTX3 plasma levels may be a better biomarker of pulmonary arterial hypertension than B-type natriuretic peptide, especially in patients with connective tissue disease [36]; whereas in acute coronary syndrome, PTX3’s diagnostic sensitivity and specificity have been shown to be higher than those of troponin T and heart-type fatty acid binding protein [37]. In sepsis, PTX3 was reported to be superior to the classical biomarkers CRP and procalcitonin (PCT), probably due to the fact that it is locally produced at the site of tissue damage or infection, rather than relying on other molecules to trigger the synthesis of body organs [31]. Also, PTX3 demonstrated advantages compared to CRP and PCT in COVID-19 patients: an early, rapid, and massive increase in the circulation preceding the clinical signs of hospital-acquired secondary infections; guiding patient management and assessment of antimicrobial treatment response, as well as its prognostic potential for adverse outcome [38]. A recent study assessed the diagnostic and prognostic value of cerebrospinal fluid (CSF) PTX3 levels in pediatric patients with different central nervous system (CNS) infections. PTX 3 showed statistically significant sensitivity in identifying bacterial versus aseptic CNS infections, as well as bacterial CNS infections compared with controls; its values were slightly lower than those of CSF lactate dehydrogenase and CSF total leucocyte count, but higher than the plasma CRP [39].

Similarities between PTX3 and CRP have led to multiple studies investigating the potential use of PTX3 as a marker for various infectious and inflammatory diseases in humans. Unlike CRP, which requires 24–30 h to reach elevated levels during an acute inflammatory response, PTX3 can rise significantly within just 6 h [22,40].

PTX3 has emerged as a significant marker of vascular inflammation and metabolic complications in diabetes. Its plasma levels correlate with chronic hyperglycemia and may serve as a more precise indicator of diabetic retinopathy (DR) and severe ketoacidosis than high-sensitivity CRP (hsCRP) [17,25]. Hyperglycemia triggers immune cells to release inflammatory mediators (including TNF-α, IL-1β, IL-6, NF-κB, TGF-β, and adhesion molecules), which further contribute to β-cell dysfunction, insulin resistance, and progression of complications such as vascular damage, neuropathy, and CVD cell loss [41]. Targeting these mechanisms may be the key to preventing complications [41].

## 3. PTX3 as a Biomarker of Inflammation in DM

The main mechanism of action of PTX3 is based on its interaction with components of the complement system, the system of plasmatic proteins that ensure the activity of antibodies and phagocytic cells in removing pathogens and damaged cells through a series of sequential steps, including phagocytosis, inflammation, and plasma membrane attack. C1q, a fraction of the classical complement C1 complex, was identified as the first ligand for PTX3, the protein binding to its globular head region via its C-terminal domain, thereby activating the complement cascade [16,20,23].

While PTX-3 is often described as a component of the innate immune response with pro-inflammatory properties, emerging evidence suggests that it also plays crucial regulatory and tissue-protective roles in various pathological conditions [22,42]. Specifically, PTX-3 has been implicated in modulating complement activation, enhancing the clearance of apoptotic cells, and promoting the resolution of inflammation [43]. In metabolic disorders, PTX-3 has been reported to exert beneficial effects by influencing adipose tissue homeostasis [44], modulating macrophage polarization towards an anti-inflammatory (M2) phenotype [45], and reducing endothelial dysfunction [46]. Furthermore, its role in vascular integrity and tissue repair underscores its potential protective functions in conditions such as obesity, diabetes, and atherosclerosis [47].

**Figure 2:**
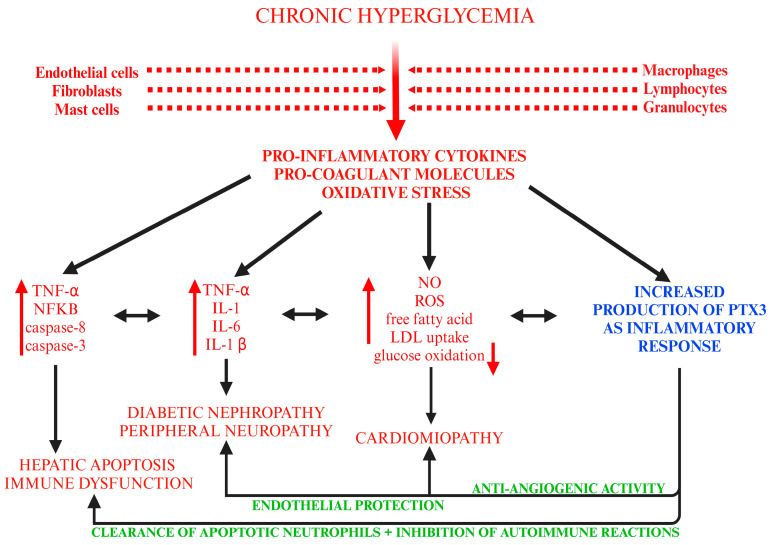
Complications determined by chronic hyperglycemia and PTX3 effects. (created with BioRender.com, accessed on 18 March 2025) (↑ elevated values; ↓ reduced values).

Chronic and inadequately treated hyperglycemia in patients diagnosed with DM can activate inflammatory responses through citokine production (TNF-α, NFkB, IL-1, IL-6, etc.) from various types of cells (such as macrophages, lymphocytes, granulocytes, fibroblasts, mast and endothelial cells). Among pro-inflammatory factors, pro-coagulant molecules and reactive oxygen species (ROS) are released, and their increase predisposes individuals to various complications (DN, peripheral neuropathy, cardiomyopathy, hepatic, and immune dysfunction). Specific citokines can determine different complications, but all of them lead to PTX3 production. PTX3 will further be able to counter the cytokine damage through protective tissular activities: clearance of apoptotic neutrophils, inhibition of autoimmune reactions, and promotion of anti-angiogenic molecules (Figure 2) [9,12,48,49,50,51].

### 3.1. Clinical Studies

#### 3.1.1. Gestational DM

Serum PTX3 levels have also been related to blood glucose and inversely associated with insulin sensitivity in pregnant women. A study conducted by Qu et al. found that a threshold of PTX3 below 1.21 pg/mL could potentially rule out gestational DM, making it a useful early indicator. The slight increase in PTX3 levels during early pregnancy can suggest its stability and potential as a reliable predictor of gestational DM progression. The involvement of PTX3 in vascular inflammation can also be linked to preeclampsia, T1DM, and future CVD risk. It has been proposed that first-trimester PTX3 levels quantification can be correlated with the later development of gestational DM, highlighting its role in the pathogenesis of DM-related complications [52,53,54].

#### 3.1.2. Diabetic Nephropathy (DN)

PTX3 is released in response to inflammatory stimuli, and its levels increase in conditions involving vascular inflammation. In DN, elevated PTX3 levels are associated with glomerular inflammation, endothelial dysfunction, and kidney injury. It may also indicate the progression of DN as it correlates with albuminuria (the presence of albumin in urine), which is a key marker of kidney damage in DM. Studies have shown that PTX3 concentrations are higher in patients with more advanced stages of DN, such as macroalbuminuria, compared to those with normoalbuminuria or microalbuminuria [55]. This suggests that PTX3 may serve as a predictive biomarker for DN progression. The ability of PTX3 to differentiate between these stages could potentially help in monitoring disease progression more accurately than other markers like hs-CRP. Since PTX3 is more sensitive than hs-CRP in distinguishing stages of DN, it holds promise as a more reliable biomarker for assessing kidney function and predicting the risk of chronic kidney disease (CKD) in diabetic patients [56]. Elevated PTX3 levels have been linked to the severity of kidney damage, and its levels may help identify patients at risk of developing end-stage renal disease [57,58,59].

Another study investigated the relationship between serum PTX3 levels and DN progression in T2DM Egyptian patients. The study included 66 T2DM patients and 22 healthy controls, with patients grouped by urinary albumin/creatinine ratio (UACR) into normoalbuminuric, microalbuminuric, and macroalbuminuric stages of DN. The results showed significantly higher serum PTX3 levels in T2DM patients compared to controls, with PTX3 levels increasing progressively from normoalbuminuric to macroalbuminuric stages. No significant difference in PTX3 was observed between controls and normoalbuminuric patients, suggesting limited utility in early DN stages. High-sensitivity C-reactive protein (hs-CRP) followed a similar pattern but was less sensitive than three in detecting DN progression, particularly in distinguishing macroalbuminuric from microalbuminuric stages. The study concluded that serum PTX3 is positively associated with DN development and progression, offering potential as a more accurate biomarker than hs-CRP for monitoring DN in T2DM patients [55].

#### 3.1.3. DR

PTX3 levels are elevated in patients with DR, reflecting the inflammatory processes in the retina. PTX3 contributes to vascular injury and permeability changes, which are key features in the pathophysiology of DR. PTX3 levels correlate with the severity of DR [60].

Hyperglycemia-induced endothelial damage initiates an inflammatory cascade that contributes to DR. PTX3, an acute-phase protein and marker of vascular injury, has proven to be significantly elevated in the aqueous humor of diabetic patients with retinopathy, especially in those diagnosed with the proliferative form of the disease. These recent findings indicate that local PTX3 production, rather than systemic glycemic control (as reflected by HbA1c levels), may play a crucial role in both the development and progression of DR [61]. Moreover, diabetic patients show higher PTX3 levels in the aqueous humor compared to non-diabetic individuals. Cytokines such as IL-1β and TNF-α, which are active in the diabetic inflammatory environment, promote PTX3 expression in various cell types, reinforcing its role at the junction of inflammation and DM. No significant PTX3 differences were found between diabetic patients with and without DR, suggesting that PTX3 elevation occurs early in the disease process and may contribute to later complications [62,63].

Higher PTX3 levels have been found in patients with proliferative DR (PDR), the most advanced stage of DR, compared to those with non-proliferative DR (NPDR). This suggests that PTX3 could be used to assess the severity of DR, helping to predict whether DR will progress from NPDR to PDR. PTX3, as a biomarker for retinal inflammation and vascular leakage, could complement traditional DR screening methods. By measuring PTX3 levels, healthcare providers might be able to identify high-risk patients earlier and make more timely interventions to prevent vision loss [26,64].

A group of researchers investigated the role of serum PTX3 as a potential biomarker for the development and progression of DR in Egyptian prediabetic and diabetic patients, to evaluate the correlation between elevated serum PTX3 levels and the presence or progression of DR. The investigation showed that serum PTX3 levels were significantly higher in patients with DR compared to those without DR, with a cut-off point of 1150 pg/mL, yielding a sensitivity of 93.3% and specificity of 72%. Similarly, hsCRP levels were elevated in patients with DR, with a cut-off point of 7.60 pg/mL, showing a sensitivity of 93.3% and specificity of 68%. The combined use of PTX3 and hsCRP reduced sensitivity to 76.7% but increased specificity to 90%. The study also found that poor glycemic control, as indicated by high HbA1c levels, was significantly associated with the development and severity of DR, emphasizing the importance of maintaining good blood sugar control. Furthermore, a longer duration of DM was linked to the progression of DR. The results suggest that serum PTX3 may be an important biomarker for DR development and severity, with elevated PTX3 and hsCRP levels correlating with DR progression. Additionally, poor glycemic control and longer DM duration are significant factors in the onset and progression of DR. This research highlights the potential of PTX3 as an early marker for DR, the value of combining biomarkers like PTX3 and hsCRP for improved diagnostic accuracy, and the importance of early screening and glycemic control in diabetic patients to reduce DR risk [65].

#### 3.1.4. Foot Ulcer

PTX3 may also be a marker for the development and severity of diabetic foot ulcers, which are another common complication of DM. Foot ulcers are often complicated by infections, and PTX3’s role in immune response and tissue repair may make it useful in predicting wound healing and inflammation. Elevated PTX3 levels might correlate with infection severity and wound chronicity, providing insight into the progression of diabetic foot complications [66].

#### 3.1.5. Cardiovascular (CV) Risk

PTX3 is involved in the pathogenesis of diabetic CV complications, such as atherosclerosis and coronary artery disease. It has been shown to play a role in vascular inflammation, which is central to the development of plaques and the progression of atherosclerosis in diabetic patients. Elevated PTX3 levels have been associated with an increased risk of CV events and may serve as a useful marker for vascular inflammation in diabetes-related heart disease. High PTX3 levels may reflect the extent of systemic vascular dysfunction, making it a potential marker for identifying DM patients at higher risk of macrovascular events, such as stroke and peripheral artery disease.

PTX3 shows significant correlations with lipids and apolipoproteins (such as ApoB, ApoC) in patients with T2DM. In both sexes, PTX3 has been associated with total cholesterol, but sex-specific differences have also been noted: in men, PTX3 correlates more with triglycerides, ApoC3, and ApoB48 (which are involved in triglyceride metabolism), while in women, it has been linked to increased values of LDL-cholesterol and ApoB100. Statistical analysis confirms that ApoC3 represents the primary factor influencing PTX3 levels in men, whereas ApoB100 plays the same role in women. In T2DM patients with non-alcoholic fatty liver disease, PTX3 has also been associated with atherogenic lipids, reinforcing its role as a marker of increased vascular risk [67,68]. In another study, in T2DM patients without non-alcoholic fat liver disease, higher PTX3 levels have been positively associated with increased waist circumference and BMI. This correlation was absent in those with non-alcoholic fat liver disease. The association between PTX3 and ApoC and ApoB in T2DM patients helps clarify the role of inflammation in the development of non-alcoholic fat liver disease and vascular complications [69].

### 3.2. Experimental Studies

Using the experimental model of streptozotocin (STZ)-induced DM, Solmaz et al. assessed the relationship between oxidative stress markers, inflammatory markers, and α-synuclein accumulation in the rat cerebellum. A total of 12 rats were used, with 6 receiving a single intraperitoneal injection of STZ (60 mg/kg) to induce DM, and 6 serving as controls. DM was confirmed 48 h later by measuring blood glucose levels. After 8 weeks, the rats were sacrificed for biochemical and immunohistochemical analysis. The results showed that plasma MDA levels were significantly elevated in the diabetic group compared to the control group, while plasma GSH levels were reduced in the diabetic rats. Plasma PTX3 levels were also significantly higher in the diabetic group than in the controls. Immunohistochemical analysis of the cerebellum revealed a significant increase in α-synuclein expression in the diabetic rats. The study concluded that oxidative stress and inflammation in chronic hyperglycemia could promote α-synuclein accumulation in the cerebellum, and that PTX3 could serve as an important biomarker for these processes [70].

Chen et al. [71] investigated the expression of PTX3 in the context of DN and its potential role in assessing renal injury and disease progression. The study aimed to evaluate whether islet transplantation, a therapeutic approach used in DM treatment, could reverse early DN and how PTX3 expression might correlate with this reversal. Previous studies had suggested that islet transplantation could effectively prevent and even reverse diabetes-induced microvascular complications like DR and DN. PTX3 is specifically detected in renal tissue, and its levels are thought to reflect renal inflammation and injury. To conduct the study, DM was induced in rats using STZ, and after 12 weeks, the diabetic rats were divided into two groups: one group receiving islet transplantation (IT) and the other serving as the DN group. Researchers assessed renal injury, renal function, and PTX3 expression through urinalysis, immunohistochemical staining, and Western blot analysis of plasma and kidney tissues. The results showed a significant decrease in PTX3 expression in the kidneys of the DN group, which was consistent with renal damage and dysfunction. However, in the islet transplantation group, PTX3 expression in the kidneys significantly increased, indicating that islet transplantation contributed to the reversal of early DN. These findings suggest that PTX3 expression in renal tissue is closely related to the extent of renal injury in DN. The study concluded that PTX3 could serve as a potential biomarker to quantify renal injury in early stages of DN, potentially allowing for earlier diagnosis and more precise tracking of disease progression [71].

Other researchers studied the role of PTX3 in DR using the STZ-induced DM model in mice. They used PTX3 knockout (PTX3KO) mice to evaluate the effects of PTX3 deficiency on DR progression. The study found that PTX3KO mice had less reactive gliosis, microglia activation, and vasoneurodegeneration in their retinas compared to wild-type diabetic mice. The PTX3KO mice also showed preserved visual function, evidenced by improved optokinetic responses and restored b-wave amplitude in electroretinograms. Additionally, the researchers observed that PTX3 was required for TNF-α-induced retinal inflammation, as retinal explants from PTX3KO mice did not show GFAP upregulation in response to TNF-α. They also found increased PTX3 levels in the retinas of diabetic mice and in human datasets, particularly in diabetic macular edema and PDR. These findings suggest that PTX3 contributes to sterile inflammation in DR, driving disease progression and visual impairment [72].

Miyaki et al. [73] investigated the role of PTX3 in insulin sensitivity, particularly in the context of obesity-induced insulin resistance. While PTX3 is known for its anti-inflammatory properties, its effect on insulin sensitivity has not been fully explored. The study focused on male diabetic–obese Tsumura Suzuki obese–diabetic (TsoD) mice and lean control mice, examining the expression levels of PTX3 and glucose transport proteins (GLUT4) in both epididymal adipose tissue and soleus muscles. The results showed that PTX3 levels were significantly lower in both adipose tissue and skeletal muscle of TsoD mice compared to the lean control group. Additionally, there was a significant positive correlation between PTX3 levels and GLUT4 expression in the skeletal muscle, suggesting a potential link between PTX3 and glucose transport regulation. These findings led to the conclusion that PTX3 may play a role in promoting insulin sensitivity in skeletal muscle, particularly in the context of obesity-induced insulin resistance in TsoD mice [73].

## 4. Evidence of the Interplay Between PTX3—DM—Antidiabetic Drugs

### 4.1. Clinical Studies

Research exploring the impact of various therapeutic interventions on PTX3 levels in diabetic patients has yielded valuable insights into its role as a biomarker of inflammation and disease progression.

A study including 106 patients with DN divided them into three groups based on estimated glomerular filtration rate (eGFR) and albuminuria levels. Group 1 included patients with eGFR > 60 mL/min and albuminuria between 30 and 300 mg/day on at least two of three measurements within the last 3 months. Group 2 consisted of patients with eGFR > 60 mL/min and albuminuria > 300 mg/day on at least two of three measurements within the last 3 months. Group 3 comprised patients with eGFR < 60 mL/min and albuminuria > 300 mg/day on at least two of three measurements within the last 3 months.

Patients in group 1 received the following antidiabetic treatments: SFN (n = 13), insulin secretagogues (n = 3), glitazones (n = 1), insulin (n = 18), metformin (n = 31), and acarbose (n = 11). In group 2, patients were treated with SFN (n = 3), insulin secretagogues (n = 2), glitazones (n = 2), insulin (n = 20), metformin (n = 20), and acarbose (n = 7). In group 3, patients received SFN (n = 3), insulin secretagogues (n = 1), glitazones (n = 2), insulin (n = 29), metformin (n = 7), and acarbose (n = 3).

PTX3 levels were measured in all groups. In group 1 (n = 37), the mean PTX3 level was 810 ± 250 pg/mL. In group 2 (n = 34), the mean PTX3 level was 940 ± 260 pg/mL. In group 3 (n = 35), the mean PTX3 level was 1350 ± 1550 pg/mL.

The authors found that PTX3 levels increased as DN progressed, correlating with the severity of renal dysfunction and proteinuria. PTX3 was suggested to be a superior inflammatory marker compared to hsCRP, as it increased in parallel with key inflammatory cytokines such as IL-1 and TNF-α. PTX3 was also associated with proteinuria independently of eGFR. Although the study did not explicitly investigate the relationship between antidiabetic medications and PTX3 levels, it is notable that insulin use increased progressively from group 1 to group 3, mirroring the increase in PTX3 levels. This observation raises the possibility that more intensive insulin therapy, often required in patients with advanced DN, could be linked to higher PTX3 levels, either as a reflection of disease severity or a potential pro-inflammatory effect of insulin. Further studies are needed to clarify the potential influence of different antidiabetic agents on PTX3 levels in patients with DN [74].

Tsurutani et al. [75] carried out research involving 270 patients with T2DM at the start of the study, with 257 patients followed up at 3 months and 211 patients completing the 12-month follow-up.

Patients received oral administration of sitagliptin at a dose of 50 mg/day for the first 3 months. After this period, adjustments were allowed, with sitagliptin doses ranging from 25 to 100 mg/day. Additionally, SFN doses were adjusted according to the Japan Diabetes Society recommendations: glimepiride ≤ 2 mg/day, glibenclamide ≤ 1.25 mg/day, and gliclazide ≤ 40 mg/day.

PTX3 levels were assessed in a subgroup of 34 patients. At baseline, the mean PTX3 level was 1880 ± 780 pg/mL. After 3 months of sitagliptin treatment, PTX3 levels significantly decreased to 1630 ± 630 pg/mL.

The authors concluded that PTX3 levels were significantly lower after 3 months of treatment with sitagliptin compared to baseline levels. This was the first study to demonstrate a reduction in PTX3 levels attributable to treatment with DPP-4i. PTX3, produced by peripheral tissues, is an acute inflammatory biomarker that reflects vascular endothelial dysfunction. The decrease in PTX3 levels suggests that sitagliptin may exert anti-inflammatory and vascular-protective effects in patients with T2DM.

These findings indicate that PTX3 may serve as an early predictive marker for the antiatherosclerotic effects of DPP-4i. Further research is necessary to confirm these results and to explore the long-term implications of DPP-4i on vascular inflammation in diabetic patients [75].

Keramat et al. [76] conducted a study involving 180 patients with T2DM, classified based on their genetic background into two groups: TT/TC genotype (n = 120) and CC genotype (n = 60).

In the TT/TC group, patients received the following antidiabetic treatments: metformin (n = 42), glibenclamide (n = 6), combination therapy with metformin and glibenclamide (n = 64), and 8 patients were without medications. In the CC group, patients were treated with metformin (n = 21), glibenclamide (n = 3), combination therapy with metformin and glibenclamide (n = 31), and 5 patients were without medications.

PTX3 levels were reported as follows: in the TT/TC group, obese patients had a mean PTX3 level of 2590 ± 380 pg/mL, while non-obese patients had a mean PTX3 level of 2760 ± 520 pg/mL. In the CC group, obese patients had a mean PTX3 level of 2440 ± 510 pg/mL, and non-obese patients had a mean PTX3 level of 2590 ± 370 pg/mL.

The authors found that PTX3 levels were significantly lower in patients with the CC genotype compared to T allele carriers after adjusting for confounding variables. These findings suggest that PTX3 levels may vary depending on genetic background and obesity status in patients with T2DM. The lower PTX3 levels observed in the CC genotype group may indicate a reduced vascular protective response in these patients [76].

In the clinical study by Moradi et al. [77], the relationship between APOA−II−265T > C polymorphism, weight loss, and PTX3 levels was investigated in 44 patients with T2DM. The patients were divided into two genotype groups: TT/TC (n = 22) and CC (n = 22). All participants followed a dietary program focused on calorie restriction and weight loss, in addition to their antidiabetic treatment.

In the TT/TC group (n = 22), the antidiabetic therapy included metformin (n = 7), glybenclamide (n = 1), a combination of metformin and glybenclamide (n = 12), and other drugs (n = 2). In the CC group (n = 22), the distribution was metformin (n = 9), glybenclamide (n = 1), metformin + glybenclamide (n = 12), and no other drugs.

Serum PTX3 levels were evaluated at three time points:

- Baseline PTX3 levels (prior to intervention) were higher in the TT/TC group (4570 ± 850 pg/mL) compared to the CC group (3820 ± 560 pg/mL).
- Before intervention, for the overall group, PTX3 was 4170 ± 710 pg/mL, with subgroup values of 4520 ± 840 pg/mL in TT/TC carriers and 3810 ± 560 pg/mL in CC carriers.
- After the dietary intervention, PTX3 levels increased significantly in the TT/TC group (5520 ± 780 pg/mL) compared to 4050 ± 400 pg/mL in the CC group, with the overall PTX3 mean level rising to 4750 ± 630 pg/mL for the entire study population.

The results suggested that improving lifestyle and reducing calorie intake may more effectively enhance PTX3 levels in T allele carriers compared to C allele carriers. However, the differences in PTX3 levels between the two genotype groups were not statistically significant. The authors concluded that, although the findings indicated potential genotype-specific responses in PTX3 regulation following weight loss, larger and longer-term studies are needed to clarify the role of APOA-II polymorphism in PTX3 modulation and its implications for inflammation in patients with T2DM [77].

The SUMMIT VIP Study was a longitudinal observational study carried out between November 2010 and June 2013, including 936 patients with T2DM. Of these, 440 patients had clinically manifest CVD (myocardial infarction, stroke, or lower extremity arterial disease), while 496 patients had T2DM without any history of CVD at baseline.

At baseline, among patients with T2DM and CVD (n = 440):

- 367 patients remained free of CV events, and the antidiabetic treatment in this subgroup consisted of metformin (n = 239), insulin (n = 108), SFN (n = 110), glitazones (n = 23), DPP-4i (n = 41), and incretin analogs (n = 20).
- 73 patients experienced a CV event, and their antidiabetic therapies were metformin (n = 45), insulin (n = 33), SFN (n = 15), glitazones (n = 2), DPP-4i (n = 3), and incretin analogs (n = 2).
- Among patients with T2DM but without CVD at baseline (n = 496):
- 464 patients remained free of CV events, with treatments including metformin (n = 331), insulin (n = 73), SFN (n = 138), glitazones (n = 33), DPP-4i (n = 52), and incretin analogs (n = 24).
- 32 patients developed a CV event, and their treatment consisted of metformin (n = 26), insulin (n = 8), SFN (n = 7), glitazones (n = 5), DPP-4i (n = 2), and incretin analogs (n = 1).

At the 3-year follow-up (N = 648), the distribution of patients and their antidiabetic treatments was reassessed:

- Among patients with T2DM and preexisting CVD (n = 327):
  ○ 276 patients had no CV event, receiving metformin (n = 175), insulin (n = 83), SFN (n = 75), glitazones (n = 13), DPP-4i (n = 37), and incretin analogs (n = 18).
  ○ 51 patients experienced a CV event, with therapies including metformin (n = 34), insulin (n = 23), SFN (n = 11), DPP-4i (n = 4), and incretin analogs (n = 1). Notably, none of the patients with CV events were receiving glitazones at follow-up.
- Among patients with T2DM but without CVD at baseline (n = 421):
  ○ 397 patients remained CV event-free, with antidiabetic therapies including metformin (n = 274), insulin (n = 86), SFN (n = 101), glitazones (n = 22), DPP-4i (n = 53), and incretin analogs (n = 23).
  ○ 24 patients developed a CV event, receiving metformin (n = 20), insulin (n = 8), SFN (n = 7), DPP-4i (n = 4), glitazones (n = 1), and none were treated with incretin analogs.

Baseline plasma PTX3 levels were assessed. Patients with preexisting CVD who experienced a CV event had higher PTX3 levels (2300 [2000–2700] pg/mL) compared to those without events (2100 [1700–2600] pg/mL). A similar pattern was observed in patients without CVD at baseline, as those who later developed a CV event had PTX3 levels of 2100 [1800–2600] pg/mL, compared to 2100 [1700–2600] pg/mL in event-free patients.

Although PTX3 levels were lower at follow-up in patients who experienced CV events compared to those who remained event-free, elevated baseline PTX3 levels were associated with a higher risk of CV complications. These results suggest that PTX3 may serve as a marker of inflammation and a predictor of CV risk in patients with T2DM, regardless of their baseline CVD status or antidiabetic treatment [78].

Another study with 75 patients investigated the relationship between serum PTX3 levels and DM status. The study population was divided into two subgroups based on their DM status. The non-diabetes group comprised 28 patients, while the diabetes group included 47 patients, of whom 43 had T2DM and 4 had T1DM. Within the diabetes subgroup, patients were receiving various antidiabetic treatments: metformin (n = 21), insulin (n = 9), SFN (n = 19), TZD (n = 4), DPP-4i (n = 11), GLN (n = 4), and AGi (n = 13). The overall PTX3 level for all patients was reported as 2810 ± 1017 pg/mL. The mean PTX3 level in the non-diabetes group was 2150 ± 1040 pg/mL, whereas in the diabetes group, the PTX3 level was higher, at 3200 ± 1910 pg/mL.

The authors concluded that serum PTX3 levels reflected chronic hyperglycemia and microalbuminuria, indicating that PTX3 could serve as a biomarker for vascular inflammation in diabetic patients. Additionally, aldosterone was identified as a novel negative regulator of PTX3 levels, providing further insight into the complex regulatory mechanisms of this inflammatory marker [79].

Bala et al. [80] conducted a study involving a total of 80 patients with T2DM who had been treated with insulin for at least six months, aiming to assess the relationship between insulin dose and inflammatory and oxidative stress markers, including PTX3.

The patients were categorized into tertiles based on their insulin dose per kilogram of body weight. The first tertile (0.18–0.57 IU/kg) included 26 patients, the second tertile (0.58–0.89 IU/kg) comprised 27 patients, and the third tertile (0.90–2.35 IU/kg) also consisted of 27 patients.

Patients in the first tertile received the following antidiabetic treatments: metformin (n = 16), SFN (n = 1), incretins (n = 2), and insulin (n = 31), with a mean insulin dose of 0.4 IU/kg/day. In the second tertile, patients were treated with metformin (n = 20), SFN (n = 2), incretins (n = 3), and insulin (n = 70), with a mean insulin dose of 0.7 IU/kg/day. The third tertile included patients receiving metformin (n = 17), SFN (n = 1), incretins (n = 2), and insulin (n = 93), with a mean insulin dose of 1.1 IU/kg/day.

The PTX3 levels in the overall sample (N = 80) were reported as 1000 ± 200 pg/mL. For each tertile, the PTX3 levels were as follows: first tertile (N = 26), 1000 ± 200 pg/mL; second tertile (N = 27), 1000 ± 300 pg/mL; and third tertile (N = 27), 900 ± 200 pg/mL.

The study found that PTX3 levels were not associated with insulin doses after adjusting for parameters known to influence inflammatory and oxidative stress markers [80].

Another study, which included 116 patients diagnosed with T2DM, evaluated the serum PTX3 levels. The study was carried out over the period 2014–2017. The mean PTX3 level in the overall cohort was 4110 ± 245 pg/mL. When stratified by sex, women (n = 49) had higher PTX3 levels (4530 ± 331 pg/mL) compared to men (n = 67), whose mean PTX3 level was 4020 ± 199 pg/mL.

Although details regarding the exact timing of PTX3 measurements within this period are not explicitly provided, the study noted that the majority of patients were receiving insulin therapy (n = 115) and/or metformin (n = 113). These findings suggest that PTX3 levels may differ based on sex in patients with T2DM, emphasizing the potential relevance of this inflammatory marker in the evaluation of diabetic populations [67].

In an observational study, 64 patients diagnosed with diabetic ketoacidosis (DKA) complicated by acute pancreatitis were assigned to two treatment groups (n = 32/group) and observed over a period of 7 days. One group received nutritional support combined with insulin therapy, while the control group received insulin therapy alone. Insulin was administered at a dose of 0.1 U/(kg·h) every 4 to 6 h in both groups.

Baseline serum PTX3 levels were comparable between groups, with the nutritional support plus insulin group having a mean PTX3 level of 9570 ± 410 pg/mL, and the insulin-only group showing 9540 ± 510 pg/mL. After 7 days of treatment, PTX3 levels were significantly reduced in both groups. The nutritional support combined with insulin therapy group exhibited lower PTX3 levels (230 ± 80 pg/mL) compared to the insulin-only group (440 ± 60 pg/mL).

The results suggest that nutritional support combined with insulin therapy is more effective than insulin alone in reducing serum PTX3 levels [81].

The study by Abaj et al. [82] analyzed the interaction between the PPAR-γ Pro12Ala polymorphism, dietary insulin load (DIL), dietary insulin index (DII), and cardio-metabolic markers, particularly focusing on patients with T2DM. The research included 393 patients, with genotype distribution as follows: 63.82% G-allele and 36.17% C-allele. All patients received metformin and glybenclamide as their primary medication regimen, while some patients were on other medications as well.

In terms of PTX3 and inflammation, the comparison between patients with the CC genotype and those carrying CG + GG genotypes revealed no significant differences in PTX3 levels, with values of 2680 ± 440 pg/mL and 2650 ± 480 pg/mL.

When analyzing PTX3 levels in relation to dietary insulin indices, the tertiles of DIL included the following number of patients: T1 (n = 131), T2 (n = 131), and T3 (n = 131). PTX3 levels across these tertiles were T1—2660 ± 430 pg/mL, T2—2710 ± 390 pg/mL, and T3—2680 ± 520 pg/mL, with a non-significant *p*-value of 0.92. Similarly, the tertiles of DII consisted of T1 (n = 131), T2 (n = 131), and T3 (n = 131) patients, with corresponding PTX3 values of T1—2690 ± 410 pg/mL, T2—2690 ± 510 pg/mL, and T3—2650 ± 410 pg/mL, also with a *p*-value of 0.92. These results indicate no apparent association between PTX3 levels and the dietary insulin indices.

Among patients receiving metformin and glybenclamide, the DIL tertiles were distributed as follows: T1 (n = 92), T2 (n = 102), and T3 (n = 118). In contrast, patients on other medications were represented as T1 (n = 18), T2 (n = 19), and T3 (n = 25). Regarding DII tertiles, patients receiving metformin and glybenclamide were distributed as T1 (n = 84), T2 (n = 112), and T3 (n = 116), whereas those on other medications accounted for T1 (n = 16), T2 (n = 15), and T3 (n = 31) patients.

Although PTX3 levels did not demonstrate significant variation based on PPAR-γ Pro12Ala genotype or dietary insulin indices, other findings within the study suggested that G-allele carriers might exhibit an increased risk of inflammation and metabolic dysfunction when adhering to insulinogenic diets. PTX3, a well-known marker of vascular inflammation and endothelial stress, has been linked to metabolic disturbances and CV risk in individuals with T2DM. While the raw data did not highlight substantial differences, the interaction analysis between genotype and diet suggested that CG/GG carriers exhibited increased PTX3 levels within the highest DIL tertile. This finding implies that genetic predisposition may amplify systemic inflammation under specific dietary conditions [82].

In a recent study conducted by Demirkilic et al. [83], patients (n = 25/group) were treated for more than three months with either metformin monotherapy or a combination of metformin and a DPP-4i. The results showed that serum PTX3 levels were higher in patients receiving the combination therapy (5470 ± 3440 pg/mL) compared to those treated with metformin alone (3790 ± 2530 pg/mL). This finding suggests a potential influence of DPP-4i on PTX3 expression, which may have implications for the inflammatory profile of patients with T2DM [83].

In a pilot study from 2024 developed by Hachuła et al. [84], 34 patients diagnosed with T2DM were treated with dulaglutide for a period of 6 months. Treatment was initiated with a dose of 1.5 mg once weekly, with an increase to 3 mg after 4 weeks in cases where adequate glycemic control was not achieved. Concomitant antidiabetic therapies included metformin (n = 36), SFN (n = 16), SGLT-2i (n = 7), DPP-4i (n = 1), and insulin (n = 9).

Serum PTX3 levels were assessed at baseline and after 6 months of treatment. At baseline, the median PTX3 level was 1288 pg/mL, with an interquartile range (IQR) of 1174.5–1381.5 pg/mL. Following the 6-month dulaglutide treatment, PTX3 levels decreased to a median of 1023.5 pg/mL (IQR: 1008–1173 pg/mL). This reduction in PTX3 suggests that dulaglutide therapy may exert anti-inflammatory effects in patients with T2DM [84].

Taken together, these studies emphasize PTX3 as a crucial inflammatory biomarker with potential clinical applications in monitoring disease progression and evaluating treatment efficacy. Various interventions, including DPP-4i, GLP-1a, and nutritional strategies, have demonstrated efficacy in reducing PTX3 levels, suggesting their ability to mitigate inflammation-associated complications in DM. Additionally, individual variability due to genetic background and gender differences appears to influence PTX3 expression, highlighting the need for more personalized treatment approaches tailored to specific inflammatory profiles. The most important characteristics of the clinical studies investigating the relationship between PTX3—DM—Antidiabetic drugs are presented in Table 1.

### 4.2. Experimental Studies

Only a few studies have concentrated on animal models, particularly rodents, to investigate the pathological significance of PTX3 and assess the impact of pharmacological interventions in diabetic conditions. These studies offer valuable insights into the molecular mechanisms driving inflammation and metabolic dysfunction, as well as the potential therapeutic benefits of various drug classes.

Artunc-Ulkumen et al. [85] analyzed the effects of exenatide on PTX3 levels in a rat model of DM. The study was conducted on female Sprague Dawley rats, divided into three groups. Group 1 (control group, n = 8) was given a normal diet without any interventions, group 2 (diabetic control group, n = 8) received an intraperitoneal (i.p.) injection of saline (1 mL/kg) as treatment, group 3 (exenatide group, 10 μg/kg/day, i.p. for four weeks, n = 8). DM was induced in groups 2 and 3 by a single i.p. injection of streptozotocin at a dose of 60 mg/kg. Control rats in group 1 were not subjected to streptozotocin treatment and were maintained on a normal palatable diet.

After the four-week treatment period, plasma PTX3 levels were measured using an enzyme-linked immunosorbent assay (ELISA) with a detection range of 78–5000 pg/mL. The mean serum PTX3 levels were as follows: group 1 (control) 1090 ± 240 pg/mL, group 2 (diabetic control) 2480 ± 210 pg/mL, and group 3 (exenatide-treated diabetic group) 1460 ± 190 pg/mL. PTX3 levels were significantly increased in diabetic rats (group 2) compared to the control group. However, exenatide treatment in group 3 significantly reduced PTX3 levels compared to the untreated diabetic group.

The results suggest that hyperglycemia and glucose toxicity in DM lead to increased PTX3 levels, reflecting systemic inflammation and potential endothelial damage. Exenatide was shown to attenuate this inflammatory response by reducing PTX3 levels, suggesting its potential role in mitigating inflammation and vascular complications associated with DM [85].

Another experimental study evaluated the effects of dapagliflozin on inflammatory and apoptotic markers, including PTX3, in a rat model of T2DM. The study involved male albino rats divided into five experimental groups: group 1 (normal vehicle group), group 2 (diabetic group), group 3 (diabetic + dapagliflozin 0.75 mg/kg), group 4 (diabetic + dapagliflozin 1.5 mg/kg), and group 5 (diabetic + dapagliflozin 3 mg/kg). T2DM was induced by feeding the rats a high-fat diet for eight weeks, followed by a single i.p. injection of STZ (30 mg/kg). The treatment with dapagliflozin was administered orally for four weeks.

The study aimed to assess the expression levels of several markers involved in inflammation and apoptosis, including PTX3. Gene expression was analyzed using polymerase chain reaction techniques. PTX3 expression was significantly downregulated in diabetic rats compared to the normal vehicle group. Treatment with dapagliflozin led to a dose-dependent upregulation of PTX3. This suggests that dapagliflozin exerts anti-inflammatory and cytoprotective effects in diabetic rats.

The findings highlight the potential of dapagliflozin not only as an antidiabetic agent but also as a modulator of inflammation and apoptosis in T2DM. The upregulation of PTX3 in response to dapagliflozin treatment suggests that PTX3 may serve as a marker of improved endothelial function and reduced inflammatory burden in DM management [86].

Both studies examined PTX3 levels as a key biomarker of inflammation and assessed the effects of two distinct antidiabetic drugs: dapagliflozin, which enhances glucose excretion through the kidneys, and exenatide, which stimulates insulin secretion and suppresses glucagon release. Despite their different mechanisms of action, both treatments showed a significant reduction in inflammatory markers, including TNF-α and PTX3. However, the exact relationship between PTX3 upregulation and inflammation control remains an area of active investigation.

## 5. Limitations

Despite the comprehensive approach of this review, several limitations must be acknowledged. One key limitation is the heterogeneity of the studies, which feature varying methodologies, study designs, and patient populations. Differences in sample size, treatment duration, and measurement techniques for PTX3 levels may contribute to inconsistencies in reported outcomes. Additionally, many studies did not account for potential confounders such as comorbidities, genetic variations, medication adherence, and lifestyle factors, which could influence PTX3 levels independently of DM progression or treatment effects.

## 6. Future Perspectives and Clinical Implications

Advancing the clinical utility of PTX3 as a biomarker for DM requires the development of standardized measurement techniques. Implementing universally accepted testing protocols could enhance study comparability, facilitating a more accurate understanding of PTX3’s role in DM progression and complications.

Understanding the diverse effects of antidiabetic medications such as GLP-1a, DPP-4i, and SGLT-2i on PTX3 levels could improve clinical decision-making. Future research should prioritize large-scale, multi-center longitudinal studies to investigate the long-term effects of pharmacological and lifestyle interventions on PTX3 expression.

Given that genetic and metabolic factors are thought to impact PTX3 regulation, personalized treatment strategies could offer more effective management of inflammation and disease progression in DM. Utilizing pharmacogenetic profiling might help tailor treatments—both pharmacological and lifestyle-related—to individual patients, improving therapeutic outcomes.

A deeper exploration of how PTX3 influences inflammation, endothelial dysfunction, and diabetic complications is necessary. Such research could reveal new therapeutic targets, particularly for cases where current treatments are insufficient or ineffective. As PTX3 plays a pivotal role in inflammation and endothelial dysfunction, developing agents that modulate its levels could lead to new treatments, either independently or in conjunction with current antidiabetic therapies.

Considering the link between PTX3 levels and DM progression, this biomarker could prove valuable for monitoring disease activity and assessing treatment effectiveness. Measuring PTX3 regularly may assist clinicians in tracking disease progression and identifying high-risk patients vulnerable to complications like DN, DR, and CVD. Targeting PTX3 as a therapeutic strategy in DM could provide new opportunities for anti-inflammatory treatments. This approach could be particularly important for patients with poorly controlled inflammation, even with adequate blood glucose levels. Directly targeting inflammation may help prevent or reduce the vascular complications frequently seen in DM.

## 7. Conclusions

PTX3 has emerged as a crucial biomarker of vascular inflammation and metabolic dysfunction in DM. Clinical and experimental studies suggest that its levels are modulated by various antidiabetic treatments, with DPP-4i, GLP-1a, and lifestyle interventions showing promising anti-inflammatory effects. However, further research is needed to elucidate the exact role of PTX3 in DM pathophysiology and its potential as a therapeutic target. Given the complex nature of inflammation in DM, a personalized approach incorporating genetic profiling and targeted pharmacological interventions may be key to optimizing disease management and preventing long-term complications.

## Figures and Tables

**Table 1:** The main characteristics of the clinical studies evaluating the interplay between PTX3—DM—Antidiabetic drugs.

Author, Year, Country	Number of Patients, Group	Gender/Age Years (Mean ± SD)		Drug (Dose)/ Number of Patients	Period of Administration	Serum PTX-3 Levels (pg/mL)	
						Before Treatment	After Treatment
Uzun, 2016 Turkey [75]	N = 106 group 1: eGFR > 60 mL/min and albuminuria between 30 and 300 mg/day on two of the three measurements within the last 3 months group 2: eGFR > 60 mL/min and albuminuria >300 mg/day on two of the three measurements within the last 3 months. group 3: eGFR < 60 mL/min and albuminuria >300 mg/day on two of the three measurements within the last 3 months.	61 female 45 male 19 female 18 male 22 female 12 male 20 female 15 male	55.88 ± 8.7 54.5 ± 10.5 54.4 ± 8.2 58.8 ± 7.3	13 SFN (-) 3 insulin secretagogues (-) 1 glitazones (-) 18 insulin (-) 31 metformin (-) 11 acarbose (-) 3 SFN (-) 2 insulin secretagogues (-) 2 glitazones (-) 20 insulin (-) 20 metformin (-) 7 acarbose (-) 3 SFN (-) 1 insulin secretagogues (-) 2 glitazones (-) 29 insulin (-) 7 metformin (-) 3 acarbose (-)	-------------------	------------------	N = 37 810 ± 250 N = 34 940 ± 260 N = 35 1350 ± 1550
Tsurutani, 2016 Japan [76]	N = 270 at the start of the study N = 257 at 3 months N = 211 at 12 months	105 female 165 male	64.3 ± 12.4	- oral administration of sitagliptin (50 mg/day13) for 3 months. - after 3 months, adjustments were allowed (sitagliptin: 25–100 mg/day). - SFN doses were adjusted according to the Japan Diabetes Society recommendations: -glimepiride: ≤ 2 mg/day -glibenclamid: ≤ 1.25 mg/day -gliclazide: ≤ 40 mg/day	12 months, PTX3 level is presented only at 3 months	N = 34 baseline: 1880 ± 780	N = 34 3 months: 1630 ± 630
Keramat, 2017 Iran [77]	N = 180 TT/TC N = 120 (obese + non-obese) CC N = 60 (obese + non-obese)	78 female 42 male 38 female 22 male	52.98 ± 6.80 56.00 ± 5.89	8 without medications 42 metformin (-) 6 glibenclamid (-) 64 metformin + glibenclamid (-) 5 without medications 21 metformin (-) 3 glibenclamid (-) 31 metformin + glibenclamid (-)	---------------	TT/TC N = 120 obese 2590 ± 380 non-obese 2760 ± 520 CC N = 60 obese 2440 ± 510 non-obese 2590 ± 370	------------
Moradi, 2017 Iran [78]	N = 44 22 people in TT/TC group metformin (n = 7) glybenclamid (n = 1) metformin+ glybenclamid (n = 12) other drugs (n = 2) 22 people in CC group metformin (n = 9) glybenclamide (n = 1) metformine+ glybenclamide (n = 12) other drugs (n = 0)	15 female/group 7 male/group	56.68 ± 5.9 56.27± 6.51 57.09 ± 5.45	metformin (-) glybenclamid (-) metformin + glybenclamid (-) other drugs (-) +dietary program	6 weeks	baseline: N = 22 TT/TC 4570 ± 850 N = 22 CC 3820 ± 560 before intervention: N = 44 4170 ± 710 TT/TC N = 22 4520 ± 840 CC N = 22 3810 ± 560	N = 44 4750 ± 630 TT/TC N = 22 5520 ± 780 CC N = 22 4050 ± 400
Shore, 2018 U.K., Italy, Sweden, Germany [79]	N = 936 at baseline: - 440 with T2DM and: - CVD (myocardial infarction, stroke, or lower extremity arterial disease): -No CV event (n = 367): glitazones (n = 23) metformin (n = 239) insulin (n = 108) SFN (n = 110) DPP-4i (n = 41) incretin analogs: (n = 20). -CV event (n = 73) glitazones (n = 2) metformin (n = 45) insulin (n = 33) SFN (n = 15) DPP-4i (n = 3) incretin analogs: (n = 2). -496 with T2DM but without clinically manifest CVD: -No CVD event (n = 464): glitazones (n = 33) metformin (n = 331) insulin (n = 73) SFN (n = 138) DPP-4i (n = 52) incretin analogs: (n = 24). -CV event (n = 32): glitazones (n = 5) metformin (n = 26) insulin (n = 8) SFN (n = 7) DPP-4i (n = 2) incretin analogs: (n = 1). N = 648 at 3-year follow-up: 327 with T2DM and: -CVD (myocardial infarction, stroke, or lower extremity arterial disease): -No CV event (n = 276): glitazones (n = 13) metformin (n = 175) insulin (n = 83) SFN (n = 75) DPP-4i (n = 37) incretin analogs: (n = 18). -CV event (n = 51) glitazones (n = 0) metformin (n = 34) insulin (n = 23) SFN (n = 11) DPP-4i (n = 4) incretin analogs: (n = 1). -421 with T2DM but without clinically manifest CVD: -No CVD event (n = 397): glitazones (n = 22) metformin (n = 274) insulin (n = 86) SFN (n = 101) DPP-4i (n = 53) incretin analogs: (n = 23). -CV event (n = 24): glitazones (n = 1) metformin (n = 20) insulin (n = 8) SFN (n = 7) DPP-4i (n = 4) incretin analogs: (n = 0).	baseline: - 440 with T2DM and: - CVD (myocardial infarction, stroke, or lower extremity arterial disease): -No CV event (n = 367): 98 female 269 male -CV event (n = 73): 25 female 48 male 496 with T2DM but without clinically manifest CVD: -No CVD event (n = 464): 164 female 290 male -CV event (n = 32) 12 female 20 male	CVD at baseline (n = 440) No CV event (n = 367) 69.4 ± 8.5 CV event (n = 73) 69.3 ± 8.7 No CVD at baseline (n = 496) No CV event (n = 464) 66.5 ± 8.7 CV event (n = 32) 68.2 ± 6.1 CVD at baseline (n = 234) No CV event (n = 200) 69.1 ± 8.4 CV event (n = 34) 71.1 ± 7.1 No CVD at baseline (n = 253) No CV event (n = 238) 64.6 ± 10.6 CV event (n = 15) 70.0 ± 7.2	glitazones (-) insulin (-) metformin (-) SFN (-) DPP-4i (-) incretin analogs (-)	November 2010– June 2013	CVD at baseline (n = 440) No CV event (n = 367) 2100 (1700–2600) CV event (n = 73) 2300 (2000–2700) No CVD at baseline (n = 496) No CV event (n = 464) 2100 (1700–2600) CV event (n = 32) 2100 (1800–2600) CVD at baseline (n = 234) No CV event (n = 200) 2000 (1700–2400) CV event (n = 34) 1900 (1700–2700) No CVD at baseline (n = 253) No CV event (n = 238) 2000 (1700–2400) CV event (n = 15) 1900 (1600–2300)	--------------------
Takashi, 2018 Japan [80]	N = 75 28 non-diabetes 47 diabetes (43 T2DM and 4 T1DM) insulin (n = 9) DPP-4i (n = 11) metformin (n = 21) thiazolidines (n = 4) sulfonylureas (n = 19) glinides (n = 4) α-GIs (n = 13)	30 female 45 male	total 55.1 ± 13.4 non-diabetes 52.4 ± 12.3 diabetes 56.8 ± 13.9	insulin (-) DPP4i (-) metformin (-) thiazolidines (-) SFN (-) glinides (-) α-GIs (-)	----------------	2810 ± 1017 2150 ± 1040 3200 ± 1910	- ---------------------
Bala, 2018 Romania [81]	N = 80 1st tertile of insulin dose/kg of body weight (0.18–0.57 IU/kg of body weight) N = 26 2nd tertile of insulin dose/kg of body weight (0.58–0.89 IU/kg of body weight) N = 27 3rd tertile of insulin dose/kg of body weight (0.90–2.35 IU/kg of body weight) N = 27	47 female 33 male 14 female 12 male 12 female 15 male 21 female 6 male	63.8 ± 9.0 65.0 ± 8.1 61.6 ± 9.9 64.8 ± 8.9	53 metformin (-) 4 SFN (-) 7 incretins (-) 70 insulin (0.7 insulin dose/kg of body weight, IU/kg/day) 16 metformin (-) 1 SFN (-) 2 incretins (-) 31 insulin (0.4 insulin dose/kg of body weight, IU/kg/day) 20 metformin (-) 2 SFN (-) 3 incretins (-) 70 insulin (0.7 insulin dose/kg of body weight, IU/kg/day) 17 metformin (-) 1 SFN (-) 2 incretins (-) 93 insulin (1.1. insulin dose/kg of body weight, IU/kg/day)	>6 months	N = 80 1000 ± 200 N = 26 1000 ± 200 N = 27 1000 ± 300 N = 27 900 ± 200	------------
Walus-Miarka, 2020 Poland [68]	N = 116 28 female + 36 male insulin 36 female + 46 male metformin	49 female 67 male	59.1 ± 11.07 62 ± 11.68 57 ± 10.17	115 insulin (-) 113 metformin (-)	2014–2017	---------------------	N = 116 4110 ± 245 N = 49 F: 4530 ± 331 N = 67 M: 4020 ± 199
Yin, 2021 China [82]	N = 64 32 nutritional support combined with insulin therapy 32 insulin	14 female 18 male 15 female 17 male	51.03 ± 5.21 50.98 ± 5.19	nutritional support + insulin (0.1 U/(kg h) once every 4 to 6 h). insulin (0.1 U / (kg h) once every 4 to 6 h).	7 days	N = 32 nutritional support + insulin 9570 ± 410 N = 32 insulin 9540 ± 510	230 ± 80 440 ± 60
Abaj, 2022 Iran [83]	N = 393 251 CC 142 CG + GG		CC 52.97 ± 6.37 CG + GG 52.81 ± 7.45	metformin + glybenclamid (-) other medications (-) tertile of DIL: metformin + glybenclamid (-) N = 131 T1 92 T2 102 T3 118 other medications (-) T1 18 T2 19 T3 25 tertile of DII metformin + glybenclamid (-) N = 131 T1 84 T2 112 T3 116 other medications (-) T1 16 T2 15 T3 31	--------------------		2680 ± 440 2650 ± 480 tertile of DIL: N = 131 T1 2660 ± 430 T2 2710 ± 390 T3 2680 ± 520 tertile of DII N = 131 T1 2690 ± 410 T2 2690 ± 510 T3 2650 ± 410
Demirkilic, 2024 Turkey [84]	N = 50 25 metformin ± DPP-4i: vildagliptin (n = 7) sitagliptin (n = 9) linagliptin (n = 9) 25 only metformin	31 female 19 male 17 female 8 male 14 female 11 male	51.38 ± 8.42 51.56 ± 9.26 51.20 ± 7.68	metformin (-) vildagliptin (-) sitagliptin (-) linagliptin (-)	>3 months	----------------------	N = 25 metformin ± DPP-4i 5470 ± 3440 N = 25 only metformin 3790 ± 2530
Hachuła, 2024 Poland [85]	N = 34 34 dulaglutide	19 female 15 male	61 ± 10.4	dulaglutide (1.5–3 mg) -treatment was initiated with a dose of 1.5 mg; patients who did not achieve significant glycemic control after 4 weeks had their dose increased to 3 mg. 36 metformin (-) 16 SFN (-) 7 SGLT2i (-) 1 DPP-4i (-) 9 insulin (-)	6 months	N = 34 dulaglutide 1288 Q1 1174.5 Q3 1381.5 Q1—first quartile; Q3—third quartile	N = 34 1023.5 Q1 1008 Q3 1173

## Abbreviations

The following abbreviations are used in this manuscript:

AGi	alpha-glucosidase inhibitors
AMP-K	activated protein kinase
Apo (ApoB, ApoC)	apolipoproteins
BGN	biguanides
BMI	body mass index
C1q	complement component
CKD	chronic kidney disease
CNS	central nervous system
CRP	C-reactive protein
CSF	cerebrospinal fluid
CV	cardiovascular
CVD	cardiovascular diseases
DII	dietary insulin index
DIL	dietary insulin load
DKA	diabetic ketoacidosis
DM	diabetes mellitus
DN	diabetic nephropathy
DPP-4i	dipeptidyl peptidase-4 inhibitors
DR	diabetic retinopathy
eGFR	estimated glomerular filtration rate
ELISA	enzyme-linked immunosorbent assay
GLN	meglitinides
GLP-1a	glucagon-like peptide-1 agonists
GLUT4	glucose transport proteins
GSH	glutathione
hsCRP	high-sensitivity CRP
IL-1β	interleukin 1β
IL-6	interleukin-6
IQR	interquartile range
IT	islet transplantation
MDA	malondialdehyde
NF-κB	nuclear factor-κB
NO	nitric oxide
NPDR	non-proliferative DR
NPTX1 and NPTX2	neuronal pentraxins
PCT	procalcitonin
PDR	proliferative DR
PPARγ	peroxisome proliferator-activated receptor γ
PTX3	pentraxin-3
PTX4	pentraxin-4
PTX3KO	PTX3 knockout
ROS	reactive oxygen species
SAP/PTX2	serum amyloid P
SFN	sulfonylureas
SGLT-2i	sodium–glucose cotransporter-2 inhibitors
STZ	streptozotocin
SUR-1	sulfonylurea receptor
T1DM	type 1 diabetes mellitus
T2DM	type 2 diabetes mellitus
TGF-β	transforming growth factor-β
TNF-α	tumor necrosis factor-α
TsoD	Tsumura Suzuki obese–diabetic
TZD	thiazolidinediones
UACR	urinary albumin/creatinine ratio
